# MRPC: An R Package for Inference of Causal Graphs

**DOI:** 10.3389/fgene.2021.651812

**Published:** 2021-04-30

**Authors:** Md. Bahadur Badsha, Evan A. Martin, Audrey Qiuyan Fu

**Affiliations:** ^1^Institute for Modeling Collaboration and Innovation, University of Idaho, Moscow, ID, United States; ^2^The Graduate Program in Bioinformatics and Computational Biology, University of Idaho, Moscow, ID, United States; ^3^Department of Mathematics and Statistical Science, Institute for Bioinformatics and Evolutionary Studies, University of Idaho, Moscow, ID, United States

**Keywords:** causal inference, graphical models, networks, principle of Mendelian randomization, gene regulatory networks, R package

## Abstract

Understanding the causal relationships between variables is a central goal of many scientific inquiries. Causal relationships may be represented by directed edges in a graph (or equivalently, a network). In biology, for example, gene regulatory networks may be viewed as a type of causal networks, where X→Y represents gene X regulating (i.e., being causal to) gene Y. However, existing general-purpose graph inference methods often result in a high number of false edges, whereas current causal inference methods developed for observational data in genomics can handle only limited types of causal relationships. We present MRPC (a PC algorithm with the principle of Mendelian Randomization), an R package that learns causal graphs with improved accuracy over existing methods. Our algorithm builds on the powerful PC algorithm (named after its developers Peter Spirtes and Clark Glymour), a canonical algorithm in computer science for learning directed acyclic graphs. The improvements in MRPC result in increased accuracy in identifying v-structures (i.e., X→Y←Z), and robustness to how the nodes are arranged in the input data. In the special case of genomic data that contain genotypes and phenotypes (e.g., gene expression) at the individual level, MRPC incorporates the principle of Mendelian randomization as constraints on edge direction to help orient the edges. MRPC allows for inference of causal graphs not only for general purposes, but also for biomedical data where multiple types of data may be input to provide evidence for causality. The R package is available on CRAN and is a free open-source software package under a GPL (≥2) license.

## Introduction

Graphical models provide a powerful mathematical framework to represent dependence among variables. Directed edges in a graphical model further represent marginal and conditional dependencies that may be interpreted as causality (Lauritzen, 1996; Spirtes et al., 2000; Koller and Friedman, 2009; Pearl, 2009; Dawid, 2010; Guyon et al., 2010; Peters et al., 2017). Directed Acyclic Graphs (DAGs), also known as Bayesian networks, are a class of graphical models with only directed edges and no directed cycles.

Multiple DAGs may represent the same conditional independencies, and therefore are Markov equivalent and belong to the same Markov equivalence class (Richardson, 1997). Without additional information, inference methods can infer only these Markov equivalence classes. For example, for a simple graph of three nodes, namely X, Y, and Z, if X and Z are conditionally independent given Y (i.e., X⊥Z | Y), three Markov equivalent graphs exist:

(1) X⊥Z|Y:X→Y→Z;X←Y←Z;X←Y→Z.

Without additional information, it is not possible to determine which graph is the truth, and the inferred graph, which represents the equivalent class, is X−Y−Z.

Existing methods for inference of DAGs or the equivalent classes fall into three broad classes (Scutari, 2010) (i) constraint-based methods (Tsamardinos et al., 2003; Kalisch and Bühlmann, 2007; Colombo and Maathuis, 2014), which perform statistical tests of marginal and conditional independence for pairs of nodes; (ii) scored-based methods (Peters et al., 2011; Mooij et al., 2016; Nowzohour and Bühlmann, 2016), which optimize the search according to a score function; and (iii) hybrid methods (Tsamardinos et al., 2006) that combine the former two approaches.

The PC algorithm (named after its developers Peter Spirtes and Clark Glymour) is one of the first constraint-based algorithms (Spirtes et al., 2000). This algorithm makes it computationally feasible to infer graphs of high dimensions, and has been implemented in open-source software, such as the R package pcalg (Kalisch et al., 2012). The R package bnlearn (Bayesian Network learn) (Scutari, 2010) implements a collection of graph learning methods from the three classes described above.Other implementations of these algorithms also exist; for example, TETRAD (A TOOLBOX FOR CAUSAL DISCOVERY), a desktop Java application (Ramsey et al., 2018).

**FIGURE 1:**
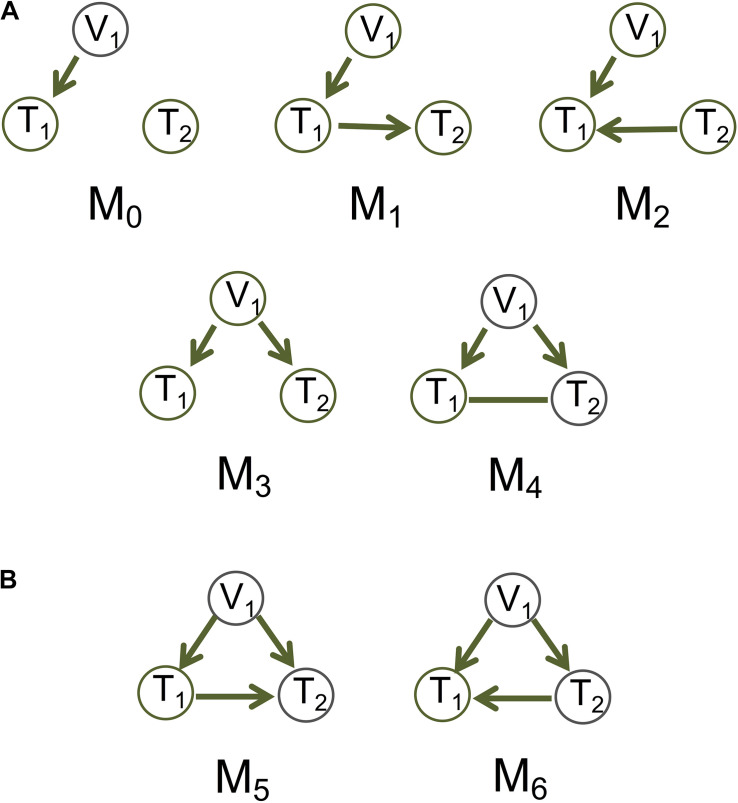
Basic causal graphs under the principle of Mendelian randomization. **(A)** The five basic (inferred) causal graphs. Each includes a genotype node (also an instrumental variable), V_1_, and two phenotype nodes, T_1_ and T_2_. **(B)** Two DAGs M_5_ and M_6_ are Markov equivalent, and can both be represented by M_4_.

The methods described above are designed for generic scenarios. In genomics there is growing interest in learning causal graphs among genes or other biological entities, with biological constraints, such as the Principle of Mendelian randomization [PMR (Smith and Ebrahim, 2003; Smith and Hemani, 2014)]. The PMR is a randomization principle that assumes the alleles of a genetic variant having been randomly assigned to individuals in a population, analogous to a natural perturbation experiment and therefore achieving the goal of randomization (Smith and Hemani, 2014). The genetic variant is then an instrumental variable that allows us to establish the causal relationship between phenotypes (e.g., gene expression). The canonical causal model (see M_1_ in Figure 1), X→Y→Z, where X is the instrumental variable, Y the exposure and Z the outcome, underlies most of the existing causal inference methods for genomic data based on the PMR (e.g., Didelez and Sheehan, 2007; Lawlor et al., 2008; Millstein et al., 2009; Smith and Hemani, 2014; Millstein et al., 2016; Wang and Michoel, 2017; Yang et al., 2017; Hemani et al., 2018; Verbanck et al., 2018; Howey et al., 2020; Zhao et al., 2020).

Whereas these methods use the genetic variant as the instrumental variable to account for *unobserved* confounding, we assume causal sufficiency, i.e., confounding variables are fully observed and may be incorporated into the network inference (Spirtes et al., 2000). We take a graphical model approach to learning causal graphs from individual-level data under causal sufficiency. For the basic models, we consider five (inferred) causal graphs involving a genetic variant node and two phenotype nodes, with the canonical model being one of them (Figure 1A and also see Figure 1 in Badsha and Fu, 2019). The PMR here means that the edges connecting a genetic variant and a phenotype always points *to* the phenotype and not the other way around. This constraint induced by the PMR provides background knowledge to the graph inference and helps limit the number of possible graphs.

Our algorithm, namely MRPC, is essentially a variant of the PC algorithm that incorporates the PMR (Badsha and Fu, 2019). MRPC implements several improvements over existing general-purpose graph inference methods, and these improvements enable us to obtain more accurate and stable inference for generic data sets compared to several methods implemented in the bnlearn and pcalg packages, both of which have been widely used for network inference. Our package further provides alternative approaches to graph visualization and graph comparison that are unavailable in the bnlearn and pcalg packages.

## Method

**FIGURE 2:**
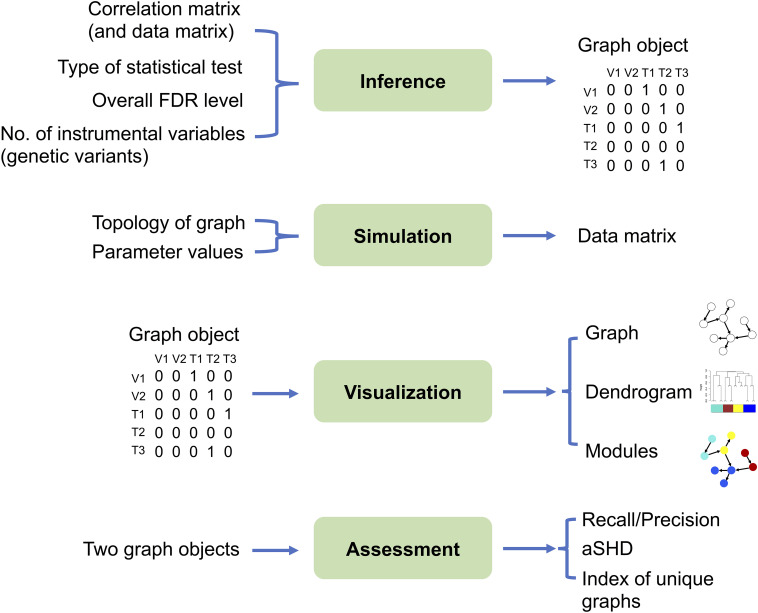
The four modules in the MRPC package. Inputs are listed on the left and outputs on the right. The inference module is at the center of the package, which may take the correlation matrix from real or simulated data as input, and outputs a graph object, the core of which is the (asymmetric) adjacency matrix. For genomic data, we require that the genotype (instrumental variable) nodes are placed in the data matrix before the phenotype nodes. Thus, the rows and columns of the correlation matrix and the adjacency matrix also start with genotypes, followed by phenotypes. The simulation module can generate a data matrix from which the correlation matrix may be derived and used as input to the inference module. A graph object, constructed directly or provided by the inference module, can be passed through the visualization module for displaying the graph topology and for clustering nodes into modules. The difference between two graph objects (e.g., true and inferred graphs, graphs inferred by two different methods) may be evaluated by multiple metrics in the assessment module.

The MRPC package contains four modules: inference, simulation, visualization, and assessment (Figure 2; two sample analysis pipelines of using these modules are provided in the Supplementary Material).

### The Inference Module

Since PC-based algorithms have demonstrated computational efficiency in learning causal graphs, we built the inference module of our MRPC algorithm on the pc function implemented in the R package pcalg. The inference module takes the data matrix or correlation matrix as input, and outputs a graph object that contains the adjacency matrix and may be visualized or compared with other graphs. The adjacency matrix of a causal graph is denoted by *A* = {*a*_*ij*_}, where *a*_*ij*_takes the value 1 if there is a directed edge from node *i* to node *j*, and 0 otherwise. In our package, we consider the rows to be parent nodes and the columns child nodes. If *a_*ij*_* = *a*_*ji*_ = 1, then the edge between nodes *i* and *j* is bidirected (which is equivalent to being undirected in our representation).

Below we describe our MRPC algorithm [first introduced in Badsha and Fu (2019)], which is at the center of the inference module. Similar to other PC-like algorithms, MRPC consists of two steps: learning the graph skeleton, and orienting edges in the skeleton:

**Step I: Learning the graph skeleton**

The procedure in this step is standard in all the PC-based algorithms: it starts with a fully connected graph, and then conducts a series of statistical tests for pairs of nodes, testing for marginal independence between two nodes, and then testing for conditional independence between two nodes, given one other node, two other nodes, and so on. An insignificant *p*-value leads to the corresponding edge being removed and never tested again in this step. The tests are similar to those in pcalg and include the Fisher’s z transformation test for Pearson correlation and partial correlation for continuous data, and the G^2^ test for discrete data (Kalisch et al., 2012).

However, pcalg and bnlearn do not control the overall error rate but only the type I error rate for each individual test. The number of total tests is also unknown beforehand: an edge removed after a test eliminates the need for additional tests for this edge. We implemented the LOND (determining the significance Levels based On the Number of Discoveries) method (Javanmard and Montanari, 2018) in order to control the overall false discovery rate (FDR) in an online manner: for each test, we use this method to calculate a *p*-value threshold. Depending on how many tests have been performed and how many rejections have been made, the *p*-value thresholds tend to be large at the beginning and decrease as more tests are performed (Badsha and Fu, 2019).

When outliers are present, MRPC further allows for the estimate of robust correlation for continuous variables (Badsha et al., 2013), which may substitute Pearson correlation.

**Step II: Edge orientation**

We design the following steps for edge orientation, which are fundamentally different from existing methods implemented in bnlearn and pcalg:

(1) If the data contain genotype information and there are edges connecting a genotype node to a non-genotype node, then the edge will always point to the non-genotype node, reflecting the assumption in the PMR that genotypes influence phenotypes, but not the other way around. An edge connecting two genotype nodes is always undirected, since it is meaningless to talk about causality between two genetic variants.
(2) Next, we search for possible triplets (X−Y−Z) that may form a v-structure (X→Y←Z), which does not have Markov equivalent graphs and can therefore be uniquely determined. We check the test results from Step I to see whether X and Z are conditionally dependent given Y. If so, then both edges are set to point to Y. Otherwise, we leave the edges undirected.

If this conditional test has not been performed in Step I (e.g., the marginal test between X and Z may result in the removal of the edge X-Z and eliminate the need for any conditional test for X and Z), we conduct it now.

If the input does not contain genotype nodes or similar instrumental variables, edge orientation will start from this step.

For undirected edges after steps (1) and (2), we look iteratively for triplets of nodes with at least one directed edge and no more than one undirected edge. We check which of the basic models in Figure 1A is consistent with the test results from Step I, and if one is found, we orient the undirected edge accordingly. It is plausible that some undirected edges cannot be oriented, and we leave them as undirected. Thus, the resulting graph may have both directed and undirected edges.

### Simulating Continuous and Discrete Data

With a known graph, we generate data first for the nodes without parents from marginal distributions, for example, a normal distribution:

(2) $$X_{i}∼N\,\left(m_{i},\sigma_{i}^{2}\right),$$

where *X*_*i*_ represents the data observed at node *i*, *m*_*i*_ is the mean and $\sigma_{i}^{2}$ the variance. If a node has one or more parents, we generate data from a conditional distribution; for example,

(3) $$X_{j}|\left\{X_{l}:l\in \text{P}\right\}∼N\,\left(\gamma_{0}+\sum_{l\in \text{P}}\gamma_{l}\,X_{l},\sigma_{j}^{2}\right),$$

where γ_0_ + ∑_*l*∈P_γ_*l*_*X*_*l*_ is the linear model that describes the dependence of *X*_*j*_ on data at its parent nodes in the set *P*.

There are different ways to simulate data of genotypes. Here we assume that each genetic variant is a biallelic single nucleotide polymorphism (SNP); this means that the variant has two alleles (denoted by 0 for the reference allele and 1 for the alternative allele) in the population. The genotype at this variant may be 0 (or 00, which means homozygous for the reference allele), 1 (or 01, heterozygous), or 2 (or 11; homozygous for the alternative allele). Let *q* be the probability of allele 1 in the population. Assume that the probability of one allele is not affected by that of the other allele in the same individual (i.e., the genotypes are in Hardy-Weinberg equilibrium). Then the genotype of the node V follows a multinomial distribution:

Pr(*V* = 0) = (1−*q*)^2^; Pr(*V* = 1) = 2*q* (1−*q*); Pr(*V* = 2) = *q*^2^.

Other types of nodes in the graph can then be simulated using the marginal and conditional normal distributions as in Expressions (2) and (3).

Different approaches may be used to generate data for graphs with an undirected edge. For M_4_ in Figure 1A, we consider that the undirected edge is a mixture of the two possible directions (Figure 1B). The nrefore, we generate data for T_1_→T_2_:

$$T_{1}∼N\,\left(\gamma_{0}+\gamma_{1}\,V_{1},\sigma_{1}^{2}\right);T_{2}∼N\,\left(\gamma_{0}+\gamma_{1}\,V_{1}+\gamma_{2}\,T_{1},\sigma_{2}^{2}\right),$$

and separately for T_1_←T_2_:

$$T_{1}∼N\,\left(\gamma_{0}+\gamma_{1}\,V_{1}+\gamma_{2}\,T_{2},\sigma_{1}^{2}\right);T_{2}∼N\,\left(\gamma_{0}+\gamma_{1}\,V_{1},\sigma_{2}^{2}\right).$$

**FIGURE 3:**
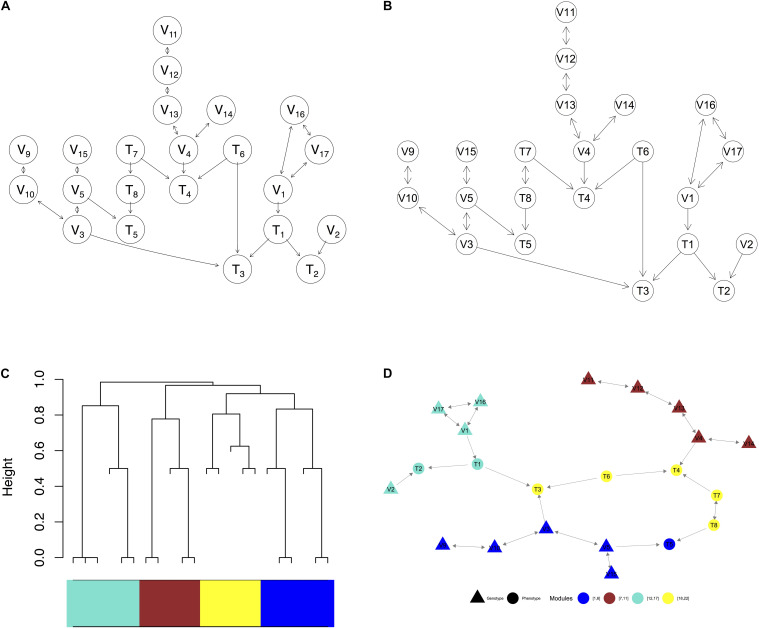
Visualization of a complex graph in MRPC. **(A)** The true graph includes 14 genetic variants and 8 phenotype nodes. **(B)** The inferred graph. **(C)** The dendrogram of the inferred graph with four modules identified when the minimum module size is set to 5. **(D)** Redrawing the inferred graph based on the dendrogram. Nodes of the same color belong to the same module.

We then randomly choose a pair of values with 50:50 probability for each sample. In a larger graph (e.g., Figure 3A), where many genotype nodes, denoted by V_*j*_, have undirected edges between them), we randomly pick a direction for each undirected edge in simulation.

For discrete data, there may exist several approaches for simulation. For the sample data in our R package, we first generate continuous values and then discretize them into multiple categories, as these sample data sets lack context and are mainly for demonstrating the usage of other functions. More appropriate methods for generating discrete data can be designed when there is more knowledge of the data generative process.

### Visualization

This module includes functions for generating three types of plots:

(a) A graph with variables represented by nodes and causal relationships by directed edges. In cases where a causal relationship is not possible to determine (e.g., see M_4_ in Figure 1A), the graph will display a bidirected edge, which is equivalent to an undirected edge.
(b) A dendrogram of all the nodes in the causal graph based on the distance (i.e., the number of edges) between two nodes. The nodes may be clustered into modules according to the dendrogram and the minimum module size, which is the number of nodes in a module.
(c) A graph with nodes in modules of different colors. Generation of this graph uses the clustering results in the dendrogram. For genomic data, the graph further displays the genotype nodes in filled triangles and phenotype nodes in filled circles.

### Assessment of Inferred Graphs

We provide three metrics to compare an inferred graph with the true one:

(a) Recall and precision: Recall (i.e., power or sensitivity) measures how many edges from the true graph a method can recover, whereas precision (i.e., 1-FDR) measures how many correct edges are recovered in the inferred graph. If an edge is recovered but its direction is wrongly inferred or not inferred, we down weigh the corresponding edge with a default value of 0.5.
(b) Adjusted Structural Hamming Distance (aSHD): The SHD, as implemented in pcalg and bnlearn, counts how many differences exist between two directed graphs. This distance is 1.0 if an edge exists in one graph but missing in the other, or if the direction of an edge is different in the two graphs. The larger this distance, the more different the two graphs are. We adjust the SHD, reducing the penalty on the wrong direction to 0.5.

### Relationship to Existing Methods and Implementations in R

We compare MRPC to the pc function from the pcalg package (Kalisch et al., 2012) and several methods implemented in the bnlearn package. Among those methods from bnlearn (Scutari, 2010), we focus on four functions: pc.stable, which also implements the same method as the function pc in pcalg; mmpc, another constraint-based method; hc (Hill Climbing), a score-based method; and mmhc, which is the hybrid version of mmpc and hc.mmhc also consists of two steps: learning the neighbors (parent and child nodes) of a node, and finding the graph that is consistent with the data and the neighbors identified from the first step (Tsamardinos et al., 2006).

There are several differences among these methods. First, although pc, pc.stable, and mmpc conduct statistical tests, they do not adjust for multiple testing and instead control the type I error rate only for each individual test. On the other hand, the default method in MRPC is the LOND method that controls the overall FDR. Second, whereas mmhc estimates a DAG with all edges being directed, the other methods considered here (including our MRPC method) estimate the Markov equivalence class of the DAG. Third, functions in bnlearn can restrict the direction of the edges involving genetic variants (with the “blacklist” argument), similar to our method under the PMR. The pc function in pcalg, however, cannot restrict only the direction of an edge but instead can include or exclude the entire edge (with the “fixedEdges” argument to include certain edges and “fixedGaps” to exclude edges).Fourth, pc and MRPC can take the correlation matrix, which is derived from the data matrix, as the input, whereas bnlearn requires the entire data matrix as the input.

## Results

### An Example

We use a graph of 22 nodes to demonstrate the functionalities of our package. Among the 22 nodes, 14 are genetic variants and 8 are phenotype nodes. Figure 3 shows the true graph, the graph inferred by MRPC, the dendrogram of the nodes, and the graph with color-coded modules identified from clustering the branches in the dendrogram. The R code for producing this figure is below:


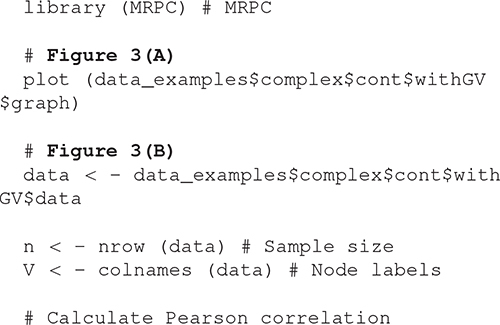



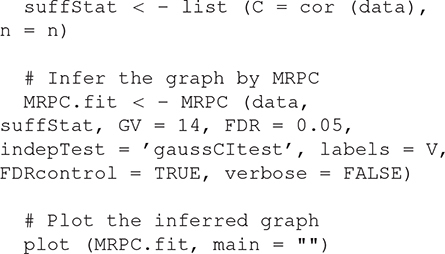


The Supplementary Material contains the R code above as well as the code for reproducing all the other analyses in Results.

### V-Structure Identification

In this and the next section, we compare our package with five implementations: the pc function from the pcalg package, and several methods (pc.stable, mmpc, hc, and mmhc) implemented in the bnlearn package. Additional simulations and method comparison may be found in our earlier work (Badsha and Fu, 2019).

Since a v-structure can be uniquely determined, it is critical to correctly identify them in the data. However, pc, hc, and mmhc may wrongly identify v-structures when there is not one. With pc, the false v-structure is due to incorrect interpretation of the lack of the edge X−Z. Specifically, with a candidate v-structure that has the graph skeleton X−Y−Z, pc examines the separation set for X and Z, denoted S(X,Z). If S(X,Z) contains Y, it means that X⊥Z | Y and X−Y−Z will not form a v-structure. When S does not contain Y, however, there may be two explanations: (i) a conditional test has been conducted for X and Z given Y, and the null hypothesis is rejected, which implies a v-structure; and (ii) the edge X−Z may have been removed due to earlier tests. As a result, the conditional test is never performed, and there is no evidence for or against a v-structure. However, pc always uses the first interpretation and claims a v-structure even when there is not one. It is unclear why hc and mmhc also falsely identify v-structures, though. To resolve the problem with pc, we have made the following improvement in MRPC in Step II (2): when determining whether a candidate triplet is a v-structure, we test conditional independence between X and Z given Y, if this test has not been performed in Step I.

To assess inference accuracy, we simulated data under Models M_1_ (not a v-structure) and M_2_ (a v-structure) from Figure 1A, and summarized the mean and standard deviation of recall and precision in Table 1. MRPC and mmpc perform similarly and do well under both models in general, although they have some problems recovering a v-structure when the signal strength is low (γ = 0.2). When the signal strength is low in the v-structure (V_1_→ T_1_←T_2_), the partial correlation between V_1_ and T_2_ conditioned on T_1_ tends to be weak and statistically insignificant. MRPC therefore tends to conclude conditional independence between V_1_ and T_2_ given T_1_, and infers an M_1_ model instead of a v-structure. hc and mmhc have lower recall than MRPC and mmpc at a weak signal strength under M_1_. pc recovers M_2_ well, and correctly infers M_1_ as V_1_–T_1_–T_2_ at moderate or strong signal. However, at weak signal, pc tends to infer a v-structure, due to the reason explained above, which results in higher recall and precision than at stronger signal, as the edge V_1_→T_1_ is now correctly inferred. Also as expected, even when we use only the edge T_1_–T_2_ to evaluate recall and precision, the metrics do not change much (see pc^∗^ in Table 1). pc.stable from bnlearn does not appear to have the same problem as pc from pcalg: pc.stable performs well under both models, with reduced accuracy only at weak signal under M_1_.

**TABLE 1:** Comparison of inference accuracy without and with a v-structure.

	Model M_1_ in Figure 1A (V_1_→T_1_→T_2_)					
	
	γ = 1.0 (strong signal)		γ = 0.5 (moderate signal)		γ = 0.2 (weak signal)	
			
	Recall	Precision	Recall	Precision	Recall	Precision
MRPC	**1.00 (0.02)**	**1.00 (0.05)**	**1.00 (0.01)**	**1.00 (0.02)**	0.96 (0.18)	**0.97 (0.12)**
pc	0.50 (0.00)	0.49 (0.03)	0.50 (0.00)	0.49 (0.04)	0.71 (0.09)	0.71 (0.10)
pc*	0.50 (0.00)	0.50 (0.00)	0.50 (0.00)	0.50(0.00)	0.50 (0.02)	0.50 (0.02)
pc.stable	0.99 (0.06)	0.97 (0.11)	**1.00 (0.05)**	0.98 (0.09)	0.77 (0.09)	0.76 (0.09)
mmpc	0.99 (0.06)	0.97 (0.11)	**1.00 (0.05)**	0.98 (0.09)	**0.97 (0.10)**	**0.97 (0.11)**
hc	0.89 (0.12)	0.78 (0.24)	0.99 (0.02)	0.99 (0.04)	0.89 (0.16)	0.90 (0.14)
mmhc	0.99 (0.03)	0.98 (0.06)	**1.00 (0.02)**	**1.00 (0.02)**	0.89 (0.16)	0.89 (0.14)

	**Model M_2_ in Figure 1A (V_1_→T_1_←T_2_)**					
	
	**γ = 1.0**		**γ = 0.5**		**γ = 0.2**	
			
	**Recall**	**Precision**	**Recall**	**Precision**	**Recall**	**Precision**

MRPC	**0.99 (0.08)**	**0.99 (0.08)**	**0.99 (0.06)**	**0.99 (0.07)**	0.73 (0.11)	0.74 (0.06)
pc	0.97 (0.12)	0.96 (0.16)	0.97 (0.11)	0.96 (0.15)	0.97 (0.13)	0.97 (0.14)
pc*	0.97 (0.10)	0.97 (0.10)	0.97 (0.11)	0.97 (0.11)	0.97 (0.11)	0.97 (0.11)
pc.stable	0.98 (0.06)	0.97 (0.12)	0.98 (0.06)	0.97 (0.11)	**0.98 (0.10)**	**0.98 (0.10)**
mmpc	0.98 (0.06)	0.97 (0.12)	0.98 (0.06)	0.97 (0.11)	0.78 (0.10)	0.77 (0.10)
hc	0.98 (0.06)	0.96 (0.13)	**0.99 (0.02)**	**0.99 (0.03)**	0.89 (0.16)	0.91 (0.13)
mmhc	**0.99 (0.06)**	**0.99 (0.07)**	**0.99 (0.02)**	**0.99 (0.04)**	0.90 (0.16)	0.91 (0.10)

Mean (with standard deviation in parentheses) of recall and precision of MPRC, pc (v2.6-2), pc.stable, mmpc, hc, and mmhc (the last four are from the bnlearn package v4.4.1) across 1,000 data sets with a sample size of 1,000 are reported. Regression coefficient γ (see Equation 3) indicates the signal strength. For the functions from bnlearn, we restricted the direction of possible edges involving the genetic variant V_1_ using the “blacklist” argument. Calculation of recall and precision uses all the edges in an inferred graph, except for the results for pc*, where edges involving the genetic variant V_1_ are excluded. Highest mean values in each column are in bold.

### Inference Accuracy of Different Methods on a Graph of 22 Nodes

We are interested in how our and other methods perform on larger graphs such as the one in Figure 3A, which contains 22 nodes and several M_1_ and M_2_ subgraphs. Similar to the previous section, we simulated 1,000 independent data sets for this graph at different signal strengths, and calculated the mean and standard deviation of recall and precision (Table 2). Since 14 of the nodes are genetic variants, we applied the blacklist argument again when running the functions from bnlearn. Recall that MRPC always infers an edge between two genetic variants to be bidirected (or undirected). Other methods, however, do not make this assumption. When calculating recall and precision for other methods, we adjusted the inferred graphs such that the direction of any edge between two genetic variants is ignored. With moderate and strong signal, MRPC and mmhc perform similarly, in mean recall and precision, both being better than other methods. MRPC further has smaller variance in recall and precision than all the other methods. When the signal is weak, mmhc and hc still perform well on both metrics. MRPC retains a high precision (higher than other methods, except for mmhc and hc), but its ability to recover the true edges is significantly reduced (i.e., lower recall). By contrast, all the other methods have higher recall but lower precision.

**TABLE 2:** Comparison of inference accuracy on the complex graph in Figure 3A.

	γ = 1.0 (strong signal)		γ = 0.5 (moderate signal)		γ = 0.2 (weak signal)	
			
	Recall	Precision	Recall	Precision	Recall	Precision
MRPC	0.98 (0.00)	**0.98 (0.00)**	0.97 (0.01)	**0.98 (0.00)**	0.66 (0.06)	0.85 (0.03)
pc	0.95 (0.01)	0.93 (0.03)	0.95 (0.02)	0.89 (0.05)	0.90 (0.04)	0.76 (0.06)
pc*	0.91 (0.04)	0.90 (0.05)	0.91 (0.04)	0.89 (0.06)	0.77 (0.11)	0.74 (0.11)
pc.stable	0.97 (0.06)	0.95 (0.04)	0.97 (0.01)	0.91 (0.05)	0.92 (0.04)	0.78 (0.06)
mmpc	0.97 (0.01)	0.95 (0.03)	0.98 (0.01)	0.91 (0.05)	0.87 (0.04)	0.74 (0.06)
hc	**0.99 (0.01)**	0.92 (0.05)	**0.99 (0.01)**	0.91 (0.05)	**0.95 (0.03)**	0.89 (0.05)
mmhc	0.98 (0.01)	0.97 (0.03)	0.98 (0.01)	0.95 (0.04)	0.94 (0.04)	**0.90 (0.05)**

See the legend for Table 1. Calculation of recall and precision uses all the edges in an inferred graph, except for the results for pc*, where edges involving the genetic variants are excluded. Highest mean values in each column are in bold.

### Node-Ordering Independence

Network inference algorithms are often sensitive to how the nodes are ordered in the input and may infer the graph differently simply because the node ordering is different. In this simulation, we generated 200 data sets for each graph with a strong signal (γ = 1.0) and a sample size of 1,000. For each data set, we permuted the order of the nodes to generate permuted data sets, applied all the methods (restricting edge direction wherever necessary and possible), and counted the number of uniquely inferred graphs. We then calculated the quantiles of the number of uniquely inferred graphs across the 200 data sets. MRPC is the most stable, whereas hc and mmhc are the most sensitive to node ordering, especially on the complex graph (Table 3). Other methods are much more stable than hc or mmhc but not as stable as MRPC.

**TABLE 3:** Summary statistics of the counts of uniquely inferred graphs with node permutation.

	V_1_→T_1_→T_2_→T_3_			
	
	1st Quartile	Median	3rd Quartile	Max
MRPC	1	1	1	1
pc	1	1	1	2
pc.stabe	1	1	1	2
mmpc	1	1	1	1
hc	2	2	3	3
mmhc	2	2	2	2

	**V_1_→T_1_←T_2_→T_3_**			
	
	**1st Quartile**	**Median**	**3rd Quartile**	**Max**

MRPC	1	1	1	1
pc	1	1	1	1
pc.stable	1	1	1	2
mmpc	2	2	2	2
hc	2	2	2	3
mmhc	2	2	2	2

	**Complex Graph in Figure 3A**			
	
	**1st Quartile**	**Median**	**3rd Quartile**	**Max**

MRPC	1	1	1	1
pc	1	2	4	39
pc.stable	1	1	1	5
mmpc	1	1	1	2
hc	42	43	45	49
mmhc	35	37	39	44

*We generated 200 data sets for each graph and then permuted the order of the nodes in each data set. We then applied the methods and counted the number of uniquely inferred graphs among the permuted data sets. We then calculated the quantiles of the number of uniquely inferred graphs across the 200 data sets.*

### Runtime

Kalisch and Bühlmann established that the computational complexity of the classical PC algorithm is at most polynomial in the number of nodes on sparse DAGs, but is exponential without the sparsity constraint (Kalisch and Bühlmann, 2007). Since MRPC uses a similar procedure to PC, the computational complexity is similar. The R implementation of MRPC builds on the pc function in the pcalg package, thus we expect that the runtime of MRPC should also be similar to pc. Additionally, whereas MRPC and pcalg are implemented in R, bnlearn implements the core functions in C, which may further reduce the runtime of the functions from bnlearn. Here, we ran each method on 1,000 independent data sets for three graphs and reported the average runtime (Table 4). As expected, MRPC and pc have similar runtime, and both of them are slightly slower than the bnlearn methods on small graphs M_1_ and M_2_. On the complex graph, all the implementations have comparable runtime, except that pc.stable is slower.

**TABLE 4:** Average runtime (in seconds) of each method for three graphs.

	MRPC	pc	pc.stable	mmpc	hc	mmhc
Model M_1_ (Figure 1A)	0.007	0.006	0.002	0.002	0.002	0.003
Model M_2_ (Figure 1A)	0.006	0.005	0.002	0.003	0.002	0.003
The complex graph (Figure 3A)	0.048	0.032	0.073	0.047	0.038	0.039

*For each graph, 1,000 independent data sets were simulated with a sample size of 500 and signal strength of 0.5.All jobs were run on a Mac computer with a CPU of 2.2 GHz and RAM of 16 GB.*

### Robustness in the Presence of Outliers

**FIGURE 4:**
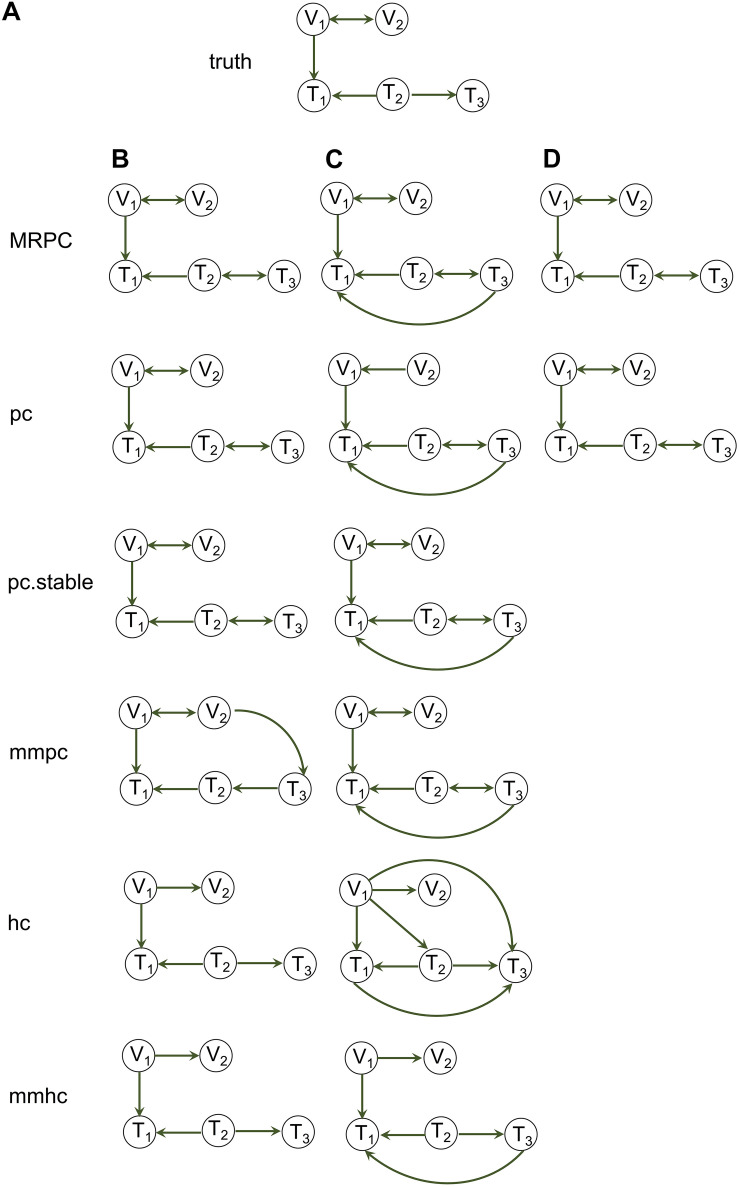
Impact of outliers on graph inference. **(A)** The true graph under which data of sample size 1,000 with and without outliers were simulated. **(B)** Inference by MRPC, pc, pc.stable, mmpc, hc, and mmhc on the simulated data that do not contain outliers, using Pearson correlation as input. **(C)** Inference by the five functions on the simulated data that contain 10 outliers, using Pearson correlation as input. In **(B)** and **(C)** the blacklist argument in pc.stable, mmpc, hc, and mmhc was used to disallow edges pointing to a genetic variant. **(D)** Inference by MRPC and pc on the simulated data with 10 outliers using robust correlation as input.

When the data are suspected to contain outliers, our MRPC package allows for calculation of a robust correlation matrix from the data (Badsha et al., 2013). Our current implementation of the robust correlation calculation is limited to continuous data for all the columns if there is no genotype information, and for the phenotype columns if there is genotype information. In the robust correlation calculation, each sample in the data matrix is assigned a weight. Outliers tend to receive a weight near 0, thus limiting their contribution to correlation. Both MRPC and pc can take the robust correlation matrix as the input for graph inference, whereas the functions in the bnlearn package do not allow for such input and therefore do not deal with outliers. As our simulation results show, when the data do not contain outliers, all the methods infer the graph mostly accurately (Figures 4A,B). However, when the data contain outliers, each of these methods infers one or more extra edges using Pearson correlation (Figure 4C). Using robust correlation, however, MRPC and pc manage to downweigh the outliers and produce a graph closer to the truth (Figure 4D).

### Dealing With Confounding in Causal Inference for Genomic Data

**FIGURE 5:**
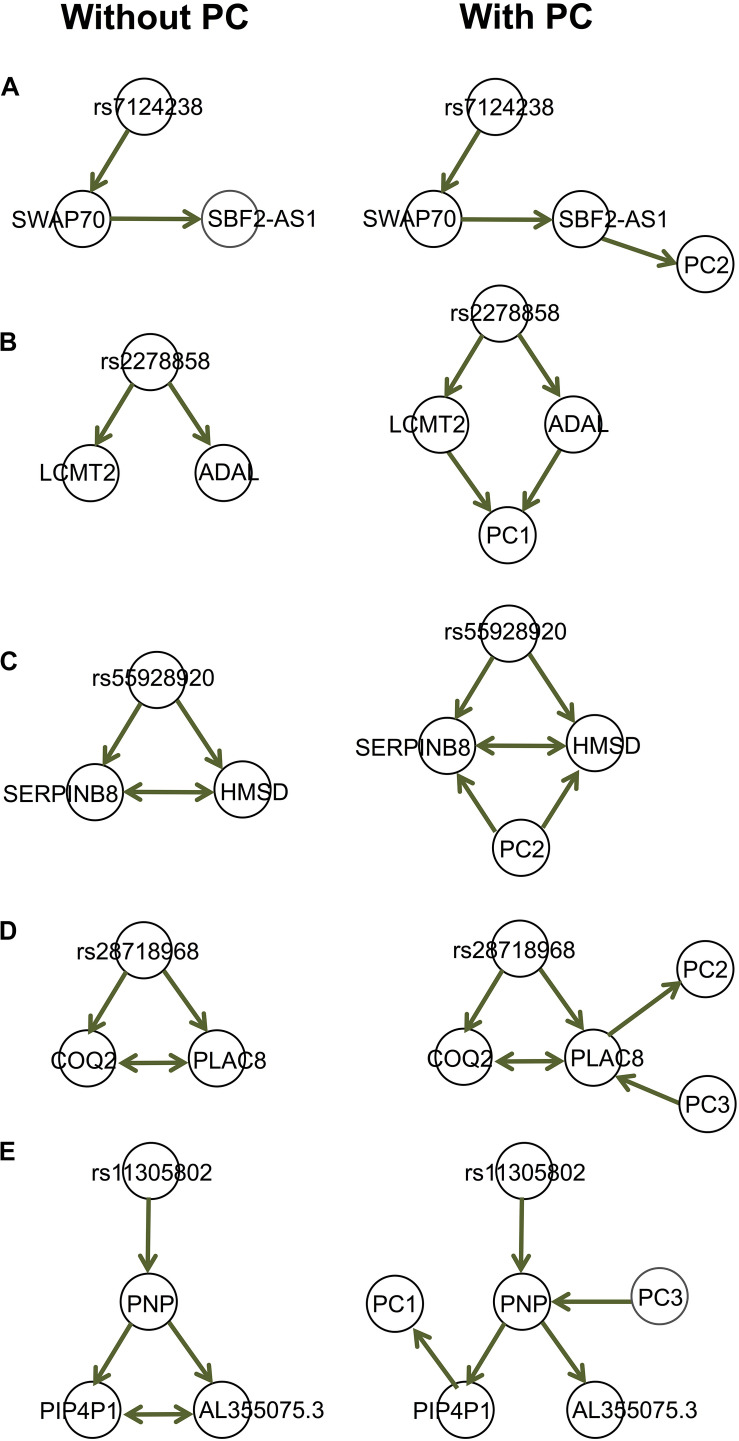
Examples of the GEUVADIS data analysis accounting for confounding variables. Each of the five sets in (**A**–**E**) contains an eQTL and multiple genes. These genes have been identified by GEUVADIS to be significantly associated with the corresponding eQTL. We derived the principal components (PCs) from the whole-genome gene expression matrix and identified the PCs that are significantly associated with the eQTLs or genes. We applied MRPC to the eQTL-gene set without and with the associated PCs. The PCs can have diverse relationships with the genes.

We provide a few examples of dissecting the regulatory relationships among multiple genes associated with the same genetic variants, accounting for (observed) confounding under the assumption of causal sufficiency [Figure 5; also see Badsha and Fu (2019)]. The gene expression data are provided by the GEUVADIS (Genetic EUropean VAriation in DISease) consortium (Lappalainen et al., 2013), which measured the genome wide gene expression levels in lymphoblastoid cell lines (LCLs) in a subset of samples from the 1,000 Genomes Project (The 1000 Genomes Project Consortium, 2015). The gene expression data have been normalized with the PEER [Probabilistic Estimation of Expression Residuals (Stegle et al., 2012)] normalization method by the GEUVADIS consortium; this method reduces potential impact from demographic variables and batch effect. We then performed Principal Component Analysis (PCA) on the genomewide gene expression matrix and extracted the top 10 PCs. The genotype data on these 373 Europeans are available through the 1,000 Genomes Project. We identified genes associated with the same expression quantitative trait loci (eQTLs), and for each eQTL-gene set, we used our function IdentifyAssociatedPCs() to identify associated PCs, using the q-value method (Storey and Tibshirani, 2003) to adjust for multiple testing. We included these PCs (FDR = 5%) as additional variables to the eQTL-gene set when we ran MRPC. The resulting causal graphs show that the PCs can have diverse relationships with the eQTL or the genes (Figure 5). The presence of the PCs did not affect the graph structure in four of the five examples here (Figures 5A–D). In the last example (Figure 5E), the edge between AL355075.3 and PIP4P1 is removed after accounting for the two PCs, although the *p*-value (0.0008) for the test is only a little larger than a rather stringent significance threshold (0.0005) with our online FDR control method.

## Discussion

Through simulation, we demonstrate that our MRPC method is stable and accurate on relatively small graphs. However, this method still needs much work for large graphs. In our earlier work (Badsha and Fu, 2019), we examined the performance of MRPC on large networks simulated in the Fifth Dialog on Reverse Engineering Assessment and Methods (DREAM5) Systems Genetics Challenge A. These networks (Net 1) contain 1,000 SNPs and 1,000 genes, each with around 2,000 edges (directed and undirected) and three different sample sizes (100, 300 and 999 individuals). We have improved the implementation of MRPC since then. However, it remains highly conservative in its inference of such large graphs and tends to infer fewer edges than there should be, similar to its performance on the 22-node graph (Table 2). On the DREAM5 networks, even at an FDR of 30%, the recall was 0.15 and the precision 0.55 for the sample size of 999, indicating that MRPC tends to retain fewer edges for which the data provide stronger evidence. MRPC performed 2–2.5 million tests on these data sets for Net 1, which took 4.6–7.5 hours on a NVIDIA Titan RTX GPU with 24 GB GPU RAM. The large number of sequential tests remains a challenge for efficiency and accuracy.

## Data Availability Statement

The source code and example data sets are available in the R package MRPC (with license GPL ≥ 2) available in the Supplementary Material and in CRAN (https://cran.r-project.org/web/packages/MRPC/index.html; official releases; standard installation) and on GitHub (https://github.com/audreyqyfu/mrpc; development; see README.md for instructions on installation).

## Author Contributions

MB developed the software. EM and AF provided improvements on the software. AF designed the project and wrote the manuscript with input from MB and EM. All authors read and approved the final manuscript.

## Conflict of Interest

The authors declare that the research was conducted in the absence of any commercial or financial relationships that could be construed as a potential conflict of interest.

## Software Information

Project name: **MRPC**

Project home page: https://cran.r-project.org/web/packages/MRPC/index.html (official releases); https://github.com/audreyqyfu/mrpc (development; see README.md for detailed instructions on installation).

Operating system(s): **Platform independent**

Programming language: **R**

Other requirements: **R 3.6.0 or higher**

License: **GPL (≥2)**

Any restrictions to use by non-academics: **GPL (≥2)**.

## Supplementary Material

Supplementary Figure 1 A sample analysis pipeline using the R package MRPC for simulating and analyzing data.

Supplementary Figure 2 A sample analysis pipeline using the R package MRPC for analyzing real data.

Supplementary Data R code for data analysis.

## Abbreviations

aSHD: Adjusted Structural Hamming Distance
bnlearn: Bayesian Network learn
CPDAG: Completed Partially Directed Acyclic Graph
DAG: Directed Acyclic Graph
DREAM5: Fifth Dialog on Reverse Engineering Assessment and Methods
eQTL: expression Quantitative Trait Locus
FCI: Fast Causal Inference
FDR: False discovery rate
RFCI: Really FCI
GEUVADIS: Genetic EUropean VAriation in DISease
GMAC: genomic mediation analysis with adaptive confounding adjustment
mmpc: Max-Min Parents and Children
mmhc: Max-Min Hill Climbing
MPDAG: Maximally oriented Partial DAG
MRPC: a PC algorithm with the principle of Mendelian Randomization
The PC algorithm: the Peter-Clark algorithm
pc: the implementation of the PC algorithm in the pcalg package
PCA: Principal component analysis
PCs: Principal Components
PEER: Probabilistic estimation of expression residuals
PMR: the principle of Mendelian randomization
SD: standard deviation
SHD: Structural Hamming Distance
SNP: Single nucleotide polymorphism
TETRAD: A TOOLBOX FOR CAUSAL DISCOVERY
TP: true positive
WGCNA: Weighted correlation network analysis for genes.
