# Supplementary figures and images for: MRPC: An R Package for Inference of Causal Graphs
Source: Front Genet. 2021 Apr 30;12:651812. doi: 10.3389/fgene.2021.651812 (PMC8120292; doi:10.3389/fgene.2021.651812)

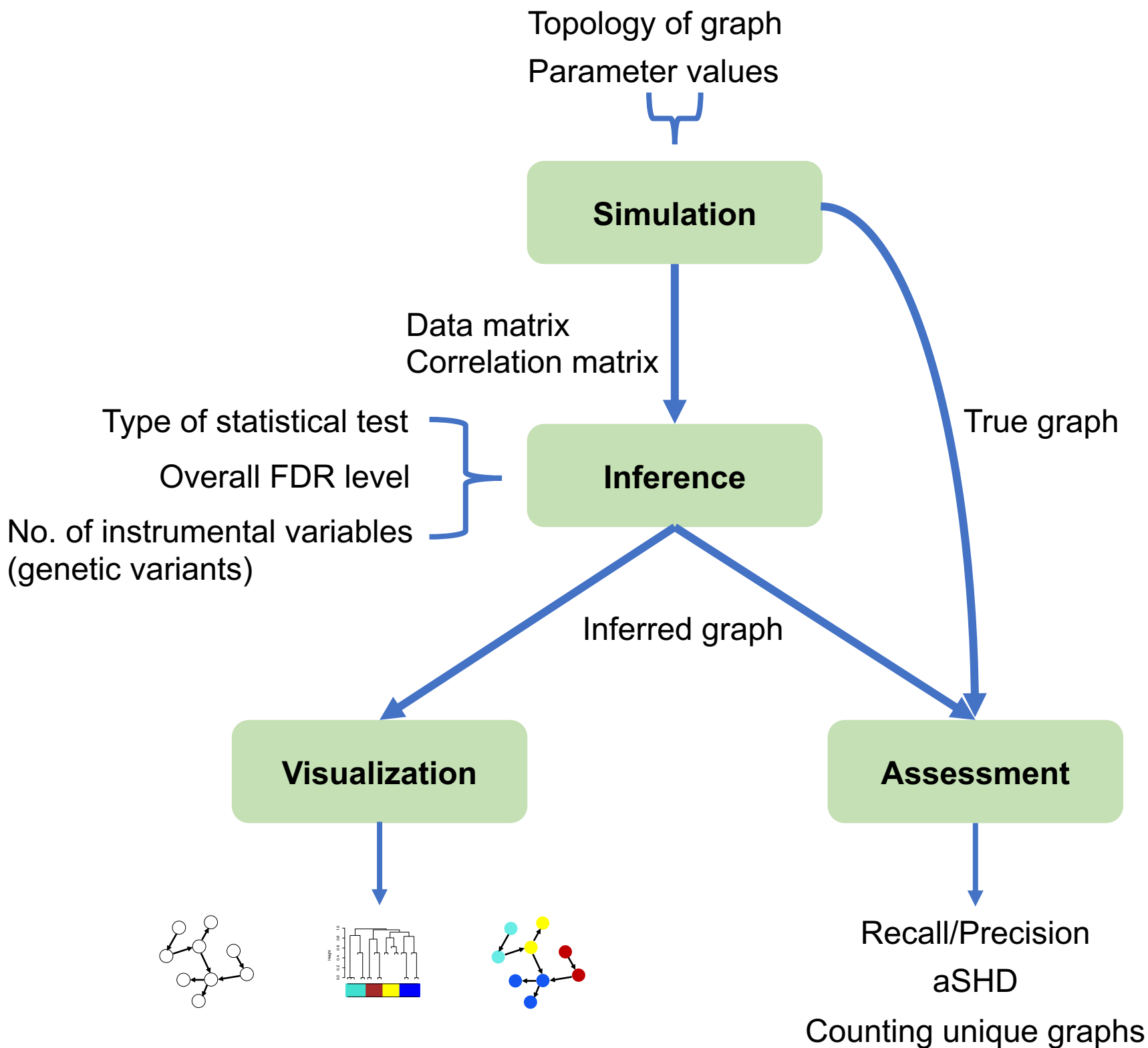

Supplement: Supplementary Figure 1 — A sample analysis pipeline using the R package MRPC for simulating and analyzing data. [file Image_1.PDF]
