# Supplementary material for: MRPC: An R Package for Inference of Causal Graphs
Source: Front Genet. 2021 Apr 30;12:651812. doi: 10.3389/fgene.2021.651812 (PMC8120292; doi:10.3389/fgene.2021.651812)
Supplement: Supplementary Figure 2 — A sample analysis pipeline using the R package MRPC for analyzing real data. [file Image_2.PDF]

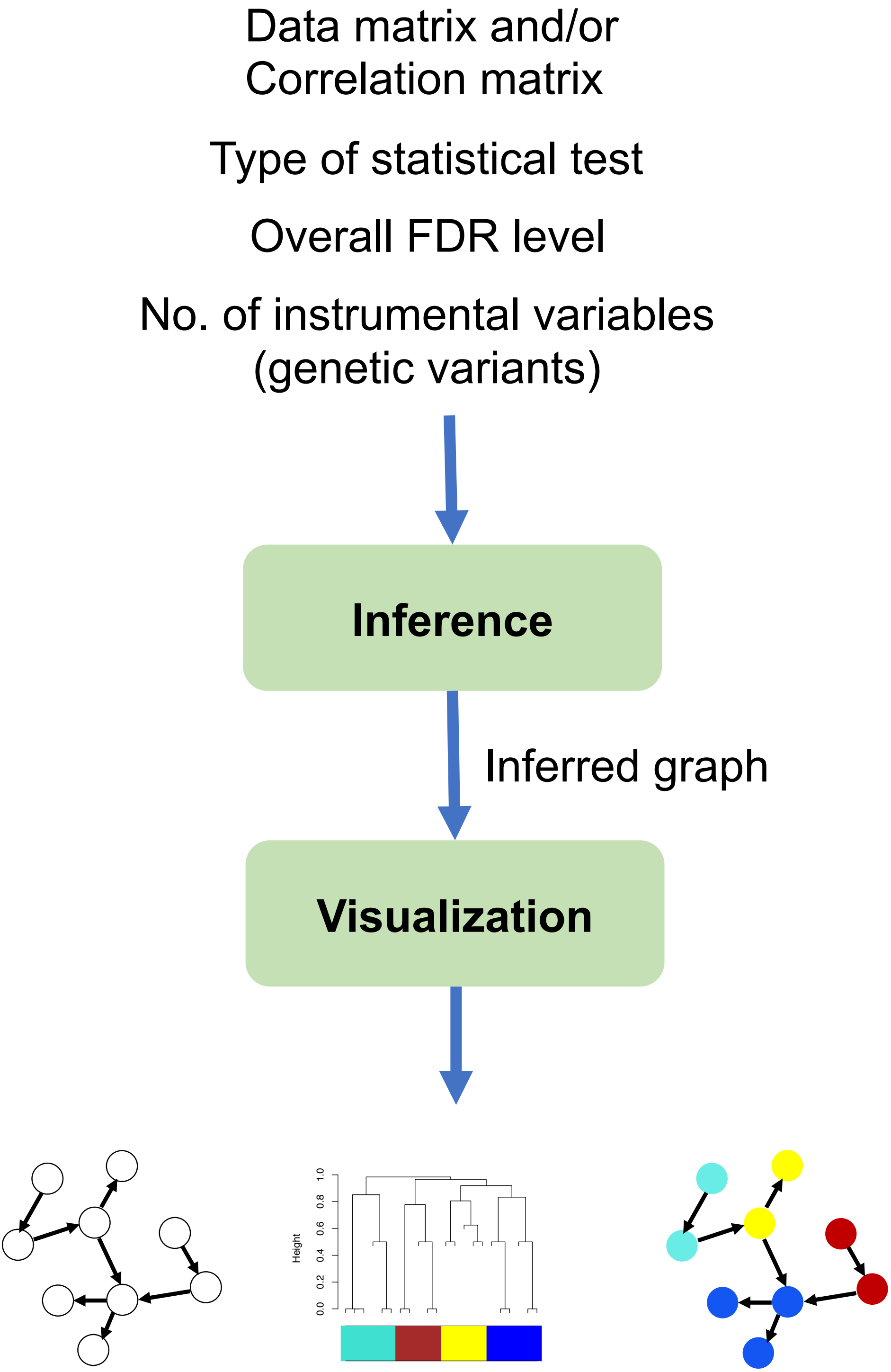
Data matrix and/or
Correlation matrix
Type of statistical test
Overall FDR level
No. of instrumental variables
(genetic variants)
Inference
Inferred graph
Visualization
Height
0.0
0.2
0.4
0.6
0.8
1.0
